# American Dental Association

**Published:** 1874-09

**Authors:** 


### Editorial.

### AMERICAN DENTAL ASSOCIATION.

The annual convocation of this body took place on the 4th to 7th of August inclusive. There was perhaps not quite the usual number of members present, but quite as many, if not more, visitors.

This meeting was an improvement on those of the last three or four years. The work progressed harmoniously throughout, there seemed to be no disturbing elements present. There were fewer special personal interests presented and pressed, than usual.

There were a number of very good papers read, upon the various subjects before the Association; and the discussions upon these were interesting and instructive. There appeared to be with almost every one a disposition to make the meeting a success. There were not reports upon all the subjects of the programme, but there were enough to profitably employ all the time of the sessions. We were gratified to see manifested a disposition to discuss more freely and fully the subject of professional education, and to suggest means, whereby, it might be improved. And it will certainly be well for those engaged as teachers, to give attention to the suggestions made; and those who assume to give direction to the preliminary education of the dental student as well.

The present membership, for the most part, consists of those who have the welfare and best interests of the profession at heart and who are willing to work assiduously to that end; and we trust that the work so well begun at this meeting will go on from year to year, till the profession shall be brought to that position, in which it shall be most efficient in the accomplishment of its proposed work.

We give on other pages of this number a synopsis of the proceedings of the first two days, the remainder will be found in the October number.

---

